# Optimization of data collection taking radiation damage into account

**DOI:** 10.1107/S0907444909054961

**Published:** 2010-03-24

**Authors:** Gleb P. Bourenkov, Alexander N. Popov

**Affiliations:** a EMBL Hamburg Outstation, c/o DESY, Notkestrasse 85b, 22607 Hamburg, Germany; b European Synchrotron Radiation Facility, 6 Rue Jules Horowitz, BP-220, 38043 Grenoble, France

**Keywords:** X-ray data collection, protein crystals, *BEST* software, radiation damage

## Abstract

To take into account the effects of radiation damage, new algorithms for the optimization of data-collection strategies have been implemented in the software package *BEST*. The intensity variation related to radiation damage is approximated by log-linear functions of resolution and cumulative X-­ray dose. Based on an accurate prediction of the basic characteristics of data yet to be collected, *BEST* establishes objective relationships between the accessible data completeness, resolution and signal-to-noise statistics that can be achieved in an experiment and designs an optimal plan for data collection.

## Abstract 2

Software implementing a new method for the optimal choice of data-collection parameters, accounting for the effects of radiation damage, is presented.

## 1. Introduction

One of the main problems in data collection from macromolecular crystals is X-ray radiation damage to the crystals. Radiation damage is the result of complex physical and chemical processes induced by absorbed X-ray photons (see reviews by Ravelli & Garman, 2006 ▶; Garman & Owen, 2006 ▶). It occurs at any temperature and leads to a resolution-dependent reduction in diffraction intensity, changes in unit-cell parameters and crystal mosaicity, slight rotations and translations of protein molecules in the lattice, disulfide-bond breaks and decarboxylation of acidic residues (Burmeister, 2000 ▶; Weik *et al.*, 2000 ▶; Ravelli & McSweeney, 2000 ▶). At cryo-temperatures a large improvement in the crystal lifetime is obtained compared with that at room temperature (Haas & Rossmann, 1970 ▶). Damage at cryogenic temperatures is a function of X-ray dose and shows no significant dose-rate dependence over the range of fluxes available at third-generation synchrotron sources (Sliz *et al.*, 2003 ▶). Radiation damage limits the information that can be obtained from a single crystal. It can also induce specific chemical modifications in the protein, which in turn can make the biological interpretations based on such an X-ray experiment problematic (Dubnovitsky *et al.*, 2005 ▶).

The effects of radiation damage must be taken into account when designing an optimal data-collection strategy, especially at third-generation synchrotron undulator beamlines, where the empirical ‘radiation dose limit for cryocooled protein crystals’ (Owen *et al.*, 2006 ▶) can be reached after a few seconds of irradiation. An incorrect choice of data-collection parameters can easily lead to failure of the experiment.

Here, we present a further development of the methods and of the computer program *BEST* (Popov & Bourenkov, 2003 ▶; Bourenkov & Popov, 2006 ▶) for optimal planning of X-ray data collection from macromolecular crystals. The strategy-determination method has been extended to take radiation damage into account. *BEST* models the statistical results of data collection based on the processing of a few initial images. The radiation-damage model in *BEST* accounts for both average intensity decay and radiation-induced non-isomorphism; model parameters common to a wide range of macromolecular structures are used in combination with the program *RADDOSE* for dose-rate calculations (Murray *et al.*, 2004 ▶) and, under the assumption that the crystal size is matched to the size of the beam, only requires a beamline with calibrated flux density. The key feature of the *BEST* strategy is compensation of the signal loss arising from overall intensity decay by a gradual increase in the exposure time.

## 2. Overview of the method

The data-collection optimization method in *BEST* (Popov & Bourenkov, 2003 ▶) is based on modelling the data statistics prior to the experiment using the information extracted from a few initial diffraction images. To a certain extent, the algorithm within *BEST* is analogous to the methods that have been developed to allow the simulation of diffraction patterns (Sarvestani *et al.*, 1998 ▶; Holton, 2008 ▶; Diederichs, 2009 ▶). A number of generalizations and approximations implemented in *BEST* make it very efficient computationally. The basic ideas are as follows.

(i) Instead of using a calculated set of diffraction intensities for a particular structure, we model them *via* the well known probability distributions derived by Wilson (1950 ▶). We denote as 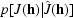 the conditional probability density function of the squared structure-factor amplitude. 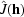 is the expectation value (the first moment) of *J*(**h**). It is a function of a reciprocal-space vector **h**. 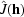 is expressed through a combination of an empirical curve defining the radial shape (the function of the resolution *h* = |**h**|, which is related to the typical interatomic distance distribution in macromolecules), the scale factor and an overall anisotropic Debye–Waller factor. The latter can be accurately estimated from a small amount of data obtained from one or two initial diffraction images.
(ii) The variance σ_*J*_^2^(**h**) associated with measurement errors is approximated by a second-order polynomial function of *J*(**h**). The polynomial coefficients *k*_0–2_ represent the error contributions of background (*k*_0_) and peak (*k*_1_) counting statistics and a systematic error (*k*_2_). These coefficients are factorized *via* a number of parameters defining the reflection condition (Lorenz and polarization factors), the crystal mosaicity, the spot shape and the background scattering distribution (extracted from the initial images) and the characteristics of the experimental setup (such as detector gain and read noise) and *via* the variable parameters of the experiment (the exposure time per frame *t*_exp_, the rotation width per frame Δ_ϕ_ and the sample-to-detector distance).
(iii) The steps analogous to simulating (with pseudo-random noise) and processing diffraction images are sub­stituted by integrating appropriate moments [*J*(**h**) and σ_*J*_^2^(**h**)] of 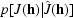 over the sampled reciprocal-space volume. This provides expressions for the expected signal-to-noise ratio in the data as a function of the data-collection parameters. Given a predefined value of the signal-to-noise ratio in a resolution shell as a target of the experiment, an optimal set of data-collection parameters is found that ensures that either the total exposure dose or the total data-collection time (including the overhead time for detector readout *etc*.) is minimized. Optimization further involves consideration of the selection of the total rotation range and the effects of the data multiplicity on the signal-to-noise ratio in the merged data. Restrictions on Δ_ϕ_ to avoid reflection overlaps are also taken into account. Thereby, both Δ_ϕ_ and *t*_exp_ are optimized for each crystal orientation (spindle position). In this way, the variation in the spatial overlap conditions is taken into account and compensation is made for the variation in scattering power arising from the anisotropic Debye–Waller factor. The resulting data-collection strategy uses few (one to five) wedges with variable exposure time and oscillation width, which is a key feature of *BEST* strategies.
(iv) Common merging statistics (such as *R* factors) are expressed analytically as functions of the signal-to-noise ratio. These *R*-­factor estimates (as well as the signal-to-noise ratios themselves) are directly comparable with the results of standard processing of data collected using an optimized (or any alternative) set of parameters.

This statistical model is based on the assumption that the crystal structure under investigation remains invariant during the experiment. This assumption is only acceptable for data collection with a low radiation dose. In the following section, we describe an extension of the statistical model of an experiment, optimization methods and formulations for apparent data statistics in the case of high-dose data collection, *i.e.* taking into account the dynamic alterations of a structure that are induced by the measurement process.

### 2.1. Radiation-damage model

#### 2.1.1. Resolution-dependent intensity decay

**Figure 1:**
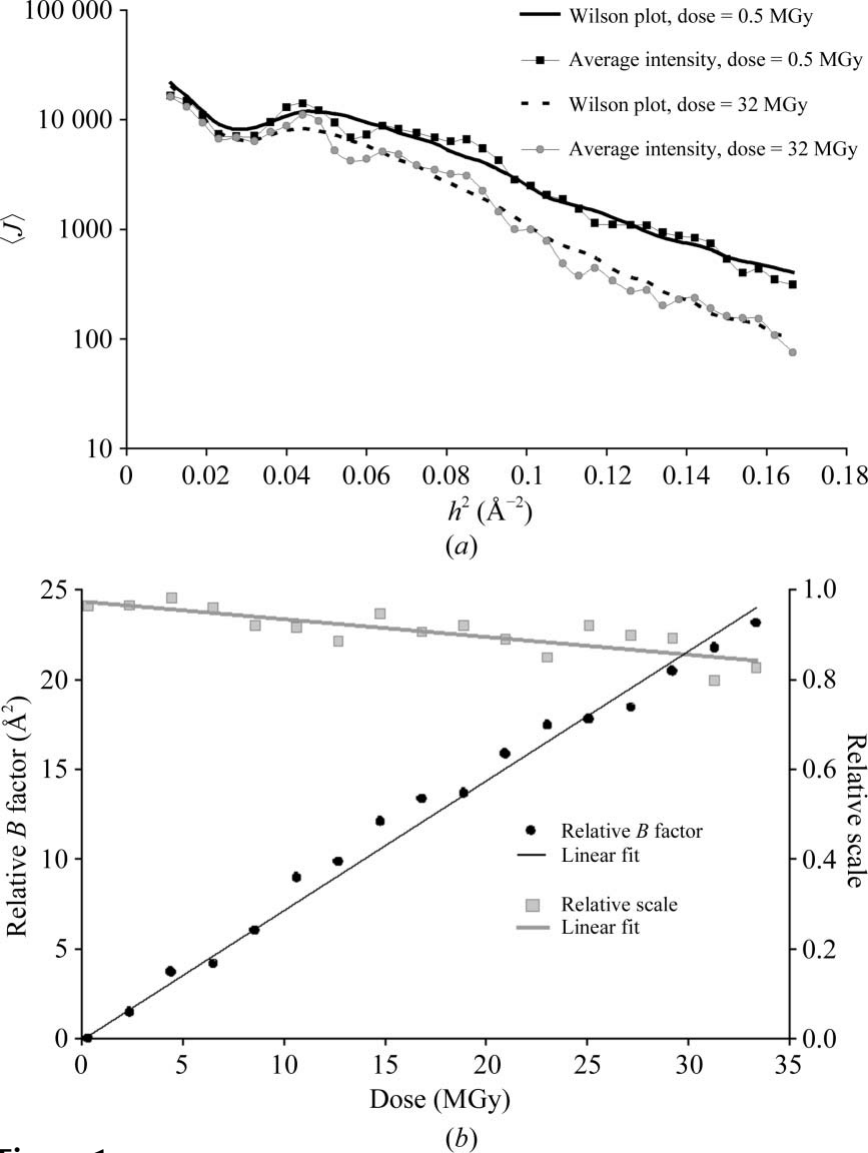
(*a*) Wilson plots (average observed *J* in resolution shells) for P19–siRNA data. Black and grey squares correspond to the fresh crystal and to the crystal after an absorbed dose of 30 MGy, respectively. The *BEST*-predicted Wilson plots, *i.e.* the average values of 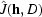 calculated for the same set of reflections, are represented by solid and dashed lines, respectively. (*b*) Relative scale and *B* factors as a function of radiation dose. Isotropic *B*-factor scaling to a common reference scale is performed by *BEST*. The scale factors are divided by those of the first data set and the *B* factor of the first data set is subtracted from the *B* factors of subsequent data sets.

The change in the scattering power after exposure to a radiation dose *D* is expressed in our model by a change in the expectation value 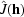. Fig. 1 ▶(*a*) shows an experimental example of its radial projection, 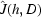, for two data sets measured from one of our test samples (P19–siRNA-1A; see §[Sec sec4.2]4.2 for experimental details) and covering the same narrow rotation range (3°) at an effectively zero dose and after an X-ray burn causing absorption of a dose *D* = 32 MGy. The total dose received by the crystal for each wedge was 0.54 MGy. Following common crystallographic methodology, the 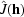 functions for a pair of isomorphous structures are related by the relative *B*-factor scaling, with the scale and isotropic *B* factor being functions of dose,
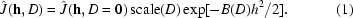
Fig. 1 ▶(*b*) shows the relative scale and *B* factors as a function of *D* determined in a series of such exposures. Here, the crystal was irradiated so that it absorbed a dose of 1.5 MGy between data collections. The example illustrates typical behaviour, characterized by a linear increase in the Debye–Waller factor *B*(*D*) = β*D*, where β is a constant scale factor representing the intensity-decay rate. Such a dependence has been observed in our systematic studies involving a large number of model structures and a variety of irradiation conditions (dose rates at different synchrotrons; Bourenkov *et al.*, 2006 ▶). The linearity of the *B*-factor increase with the dose has been confirmed in an independent study by Thorne and coworkers (Kmetko *et al.*, 2006 ▶). Moreover, the decay rates observed in these two investigations of β ≃ 1 Å^2^ MGy^−1^ are also in very close agreement. These results are furthermore in good agreement with the linear decay of the net diffraction intensity in a broad resolution shell (*h* ^−1^ > 2.5 Å) to 50% after a radiation dose of 43 MGy observed by Garman and coworkers (Owen *et al.*, 2006 ▶), despite differences in the details of the data analysis. To relate this ‘radiation dose limit for cryocooled protein crystals’ to the *B*-factor decay model, it is sufficient to integrate the 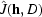 function over a corresponding resolution shell. An extensive discussion unifying many observations supporting this model is given by Holton (2008 ▶).

In addition, it is worth noting that the increase in the Debye–Waller factor accounts for more than a tenfold decrease in the scattering power at *D* = 32 MGy and *h* ^−1^ = 2.5 Å, whereas the change in the relative scale factor is responsible for a decrease of less than 20%. Presumably, the variation in scale factor can be neglected in a statistical model which aims to optimize the collection of high-resolution data.

#### 2.1.2. Radiation-induced non-isomorphism

Similar to classical *B*-factor scaling, radiation-induced non-isomorphism can be described by means of the well known Luzzati model (Luzzati, 1953 ▶). The non-isomorphism between two closely related structures, in our case one fresh and one irradiated to absorb a dose *D*, is modelled by a standard resolution-dependent non-isomorphism parameter σ_A_ (Read, 1986 ▶). We denote σ_B_(**h**, *D*) as an expected absolute difference between reflection intensities at different doses 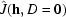 and 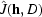, 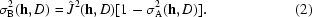
Appropriate renormalization (scaling) of the ‘damaged crystal’ data by a factor 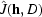 is assumed.

In our model, σ_A_ is expressed as an exponential function of both the dose and the resolution, σ_A_(*h*, *D*) = exp(−α*Dh* ^2^/4). The exponential dependence of σ_A_ on the resolution has a direct analogy with methods of σ_A_ modelling in structure refinement and phasing (*e.g.* Murshudov *et al.*, 1997 ▶; de La Fortelle & Bricogne, 1997 ▶), where the representation of σ_A_ by a single exponential (as well as *B*-factor scaling) simply corresponds to the assumption that it is the same number of atoms in both structures that are being related. This assumption holds rather well in our case. The linearity with dose and quantification of the decay parameter α are substantially more difficult to demonstrate experimentally (compared with that shown in the previous section for *B* factors). This is because the variance represented by σ_A_ is always strongly convoluted with experimental errors and separating the two contributions requires rather elaborate data analysis. We have carried out such an analysis on a large number of model structures (Bourenkov *et al.*, 2006 ▶), but the details are beyond the scope of this paper and will be published elsewhere.

For a pair of redundant or symmetry-equivalent observations recorded after absorbed doses *D*_1_ and *D*_2_, we define an exponential model of the correlation coefficient as a function of dose and resolution,
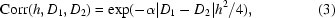
which expresses, given a small value of the parameter α, our expectation that for a small increment the two observations will show small radiation-induced differences from each other.

### 2.2. Optimization of data collection

#### 2.2.1. Signal-to-noise dependence on dose

**Figure 2:**
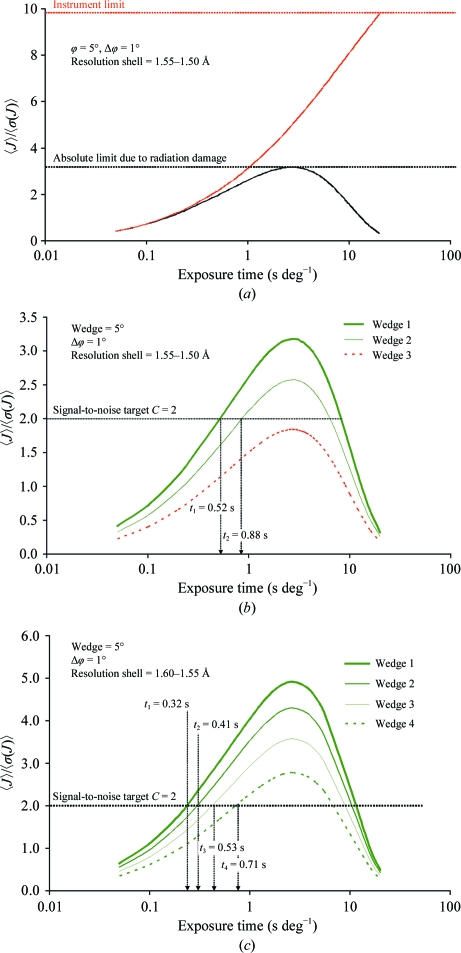
Modelling the data statistics for a cubic insulin crystal. (*a*) The signal-to-noise ratio, 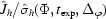, at a resolution *h* ^−1^ = 1.5 Å for five sequential frames of Δ_ϕ_ = 1° and a |Φ| = 5° wedge of data *versus* the exposure time per frame. The calculations were carried out with (black line) and without (red line) accounting for radiation damage. (*b*) Signal-to-noise ratio and graphical solution of a set of equations (§[Sec sec2.2.2]2.2.2) at a resolution of 1.5 Å for three progressive subwedges. The signal-to-noise target was *C* = 2, Δ_ϕ_ was fixed at 1° and |Φ| = 5°. No solution was possible for the third subwedge. (*c*) The same as (*b*) for four consecutive wedges and resolution 1.55 Å.

Let us consider a rotation interval (wedge) Φ of data measured with a con­stant *t*_exp_ and Δ_ϕ_ at a dose rate ρ_*D*_ (in Gy s^−1^). The width of this interval, |Φ|, is chosen to be small compared with the rotation range required for a complete data set but substantially broader than the integration range of a single reflection (*e.g.* |Φ| ≃ 5°). The expected value of the intensity of a reflection **h** observed at a spindle position ϕ ∈ Φ, with β being the intensity-decay parameter defined above, is given by
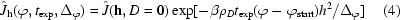
and the expected value of its standard uncertainty is
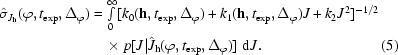
Averaging 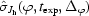 and 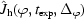 for a list of reflections predicted at Φ, one obtains an expected value of the signal-to-noise ratio for a resolution shell *h* as a function of exposure time and rotation range per frame, 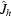/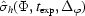. Fig. 2 ▶(*a*) represents an example of such a function of exposure time (Δ_ϕ_ = 1° is fixed) modelled for a crystal of cubic insulin (see §[Sec sec4.1]4.1 for experimental details). For comparison, the same model is shown for the hypothetical case of ρ*_D_* = 0. Neglecting the radiation damage, the maximum attainable signal-to-noise ratio is limited by the contribution of the instrumental error (*k*_2_) or by the dynamic range of the detector. For this example, with an exposure time of *t*_exp_ ≥ 20 s no data could be collected at a resolution *h* ^−1^ ≥ 1.5 Å owing to detector overload. The radiation damage sets an absolute limit on the statistics of 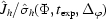 ≤ 3.5, which could be attained using an optimal *t*_exp_ = 2.5 s per 1° rotation (for a given interval but not for a complete data set). It is obvious that the pattern in Fig. 2 ▶(*a*) would shift monotonically downwards and to the right for higher resolutions [smaller, faster decaying 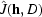 and shorter exposures] and *vice versa* at lower resolutions.

#### 2.2.2. Formulation of the optimization problem

Let us further assume that the rotation range providing a complete data set is chosen and partitioned into a series of consecutive subwedges Φ*_i_*. Optimizing the data collection then means searching for a set of exposure parameters {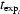, 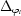} that satisfy a set of simultaneous equations
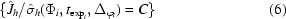
at a highest possible resolution *h* = *h*_max_(*C*). The statistical signal-to-noise target *C* must be chosen according to the crystallographic problem being addressed. The choice of *C* typically accounts for the data multiplicity given by the choice of rotation interval (assuming that the signal-to-noise ratio in a complete data set will be inversely proportional to the square root of the multiplicity).

#### 2.2.3. The algorithm

The solution is found iteratively *via* a highly efficient computational procedure. For a first trial, a high value of *h* is selected such that no solution to (6) is possible even for a first subwedge (the requested signal-to-noise ratio is above the maximum). *h* is decremented by a small step until the solution {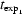, 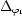} in a first subwedge is found. As can be seen from Fig. 2 ▶, the solution is not unique and, obviously, the solution with the highest speed of rotation 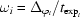 (and hence with the lowest radiation dose) is selected. The constraints on 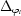 which are set by reflection spatial overlaps are taken into account. The expected decrease in scattering power induced by the dose *D*_1_ = ρ*_D_*|Φ|/ω_1_ accumulated while collecting the first wedge is then considered by substituting 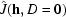 by 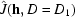 in (4) before the iteration proceeds to a second wedge. There  the solution (if it exists) will again be found, typically with a slower rotation and a higher dose *D*_*i*+1_ > *D_i_* required *etc*. Thus, the optimization problem is solved by decrementing the resolution until the *h*_max_ is found at which the solution to (6) exists for the last subwedge.

Figs. 2 ▶(*b*) and 2 ▶(*c*) illustrate an optimization procedure for the above example of insulin. The full required interval of 20° was split into four subwedges. *C* = 2 was selected as an optimization target. Only the first two subwedges could be measured with the required signal-to-noise ratio at a resolution of 1.50 Å. A solution does not exist for a third subwedge. However, a solution does exist for all four subwedges at a resolution of 1.55 Å.

### 2.3. Predictive merging statistics

The quality and internal consistency of the data sets are characterized by statistics expressing the variation of multiple (redundant and symmetry-equivalent) observations with respect to their σ^−2^-weighted average. Let us consider a set of *m_hkl_* such observations *J*_**h***j*_^*o*^ of a unique reflection *hkl* observed at respective dose values *D*_**h***j*_ and rotation speeds ω_**h***j*_. The expected standard uncertainties 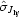 are obtained by substituting the dose rate and measurement conditions into (4) and (5). If frame-to-frame scaling uses the first frame in the data set as a reference, an expected scale factor applied to the *j*th observation is approximately *s*_**h***j*_ ≃ exp(−β*D*_**h***j*_)ω_1_/ω_**h***j*_, where 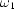 is the rotation speed of the first subwedge. Denoting 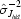 = 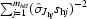 and expanding standard equations for σ^−2^-weighted merging (*J_hkl_^o^* = 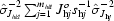), it is easy to show that the variance of *J*_**h***j*_^*o*^ *s*_**h***j*_ about *J_hkl_^o^* is expressed by 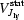 = 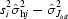. Note that 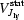 only accounts for statistical measurement errors in the data.

Another independent term that contributes to the above variance originates from radiation-induced non-isomorphism. Following similar considerations for statistical variance and constructing a covariance matrix for a set of observations with considerations according to (2) and (3) one obtains (omitting straightforward derivation)
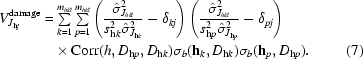
Here, δ*_ij_* is a Kronecker delta.

The expected value of *R*_merge_ is then approximated to
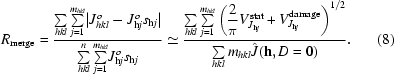

            

The multiplier 2/π reflects the fact that 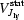 is the variance of a sample from a normal distribution (measurement errors), whereas 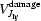 is associated with an exponential distribution (see, for example, Srinivasan & Parthasarathy, 1976 ▶). The function 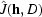 obeys the metric point symmetry of the crystal.

Finally, the average signal-to-noise in the merged data, 〈*J*/σ(*J*)〉, which is usually estimated in data processing after applying some fudge factors correcting for unaccounted radiation-induced variance, is approximated by 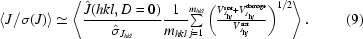
Estimations according to (8) and (9), computed by summation over unique *hkl* in either the resolution shells or for a data set, are directly comparable with the respective values obtained from data processing.

## 3. Implementation

The above formulations were implemented in the program *BEST* (versions 3.0 and higher). *BEST* uses as input the results (the basic crystallographic parameters and integrated intensities) of the processing of the initial images by *HKL* (Otwinowski & Minor, 1997 ▶), *MOSLFM* (Leslie, 1992 ▶) or *XDS* (Kabsch, 1993 ▶). The background scattering pattern is obtained from the *MOSFLM* or *XDS* output or evaluated by *BEST* directly from the diffraction images. For the radiation-damage model the only required parameter is a dose rate. In the current implementation the parameters of the decay model α and β are fixed at 0.1 and 1.0 Å^2^ MGy^−1^, respectively.

The optimization process begins by finding the shortest rotation range that provides a complete data set for starting at ϕ = 0. The statistical signal-to-noise target of 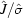 in the highest resolution shell defined by the user is divided by the square root of the multiplicity in this interval to obtain the optimization constant *C* (6). Thus, the user request is related to the statistics of a complete data set. Note that for the sake of computational efficiency the optimization target 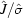 is different from, although very similar to, the 〈*J*/σ(*J*)〉 signal-to-noise statistic that is used for judging the final data quality. The rotation range is partitioned into narrow (2–5°) subwedges and optimization is carried out as outlined in §[Sec sec2.2]2.2, which results in determination of the attainable resolution *h*_max_(*C*) and an associated set of {*t*_exp_, Δ_ϕ_} pairs. The procedure is repeated for all starting angles in steps of 1°. The rotation interval that provides the highest attainable resolution is then again extended while *h*_max_(*C*) increases. Thus, both the starting angle of data collection and the multiplicity are optimized. The implementation allows the application of a variety of constraints, for example on the rotation interval, the minimum acceptable multiplicity or Δ_ϕ_, the total dose or total time of an experiment. The maximum resolution may also be constrained (to a value below an attainable resolution). In this case, the rotation interval is chosen using a minimum-dose criterion.

In order to simplify the practical implementation of this multi-subwedge data-collection strategy with currently available data-collection interfaces, as well as further data reduction with available software, the small subwedges are appropriately recombined into a few (typically 3–6) larger subwedges of variable length. Thereby, insignificant differences in the optimal *t*_exp_ and Δ_ϕ_ between the adjacent small subwedges are smoothed out. This final data-collection strategy, consisting of a data-collection resolution (*i.e.* the detector distance) and a set of quadruples {ϕ_start_, number of frames, *t*_exp_, Δ_ϕ_} is presented to the user as a final solution, together with a set of expected standard data statistics comprising completeness, multiplicity, *R*_merge_, 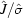 and 〈*J*/σ(*J*)〉 in the resolution shells.

## 4. Testing

In the following section, experimental examples are presented that demonstrate the validity of the approach. All measurements were carried out at the European Synchrotron Radiation Facility (ESRF, Grenoble, France) on beamline ID23-1 (Nurizzo *et al.*, 2006 ▶). The detector was an ADSC Q315. The X-ray beam profile at ID23-1 has a Gaussian shape, with FWHM (full-width half-maximum) dimensions of 30 µm vertically and 40 µm horizontally at the sample position. The incident-beam intensity was monitored continuously and the monitors were calibrated to an absolute scale (photons s^−1^) over the whole energy range. The exposure time per image at ID23-1 was not shorter then 0.1 s; in cases where shorter exposures were needed the beam was attenuated. An exposure time of 0.1 s and a rotation width of 1° were used for collecting initial images in all experiments

The program *RADDOSE* (Murray *et al.*, 2004 ▶) was used to estimate the absorbed dose on the basis of structure com­position and crystallization conditions as indicated in the literature reference for each of the samples (except for FtsH). *MOSFLM* (Leslie, 1992 ▶) was used to process both the initial images and the collected data sets and *SCALA* (Evans, 2006 ▶; Collaborative Computational Project, Number 4, 1994 ▶) was used for scaling and evaluating the data statistics. For com­parison of predicted and observed intensity-decay curves, the resolution-dependent scale factors *versus* frame number were extracted from the *SCALA* output.

### 4.1. Insulin

**Figure 3:**
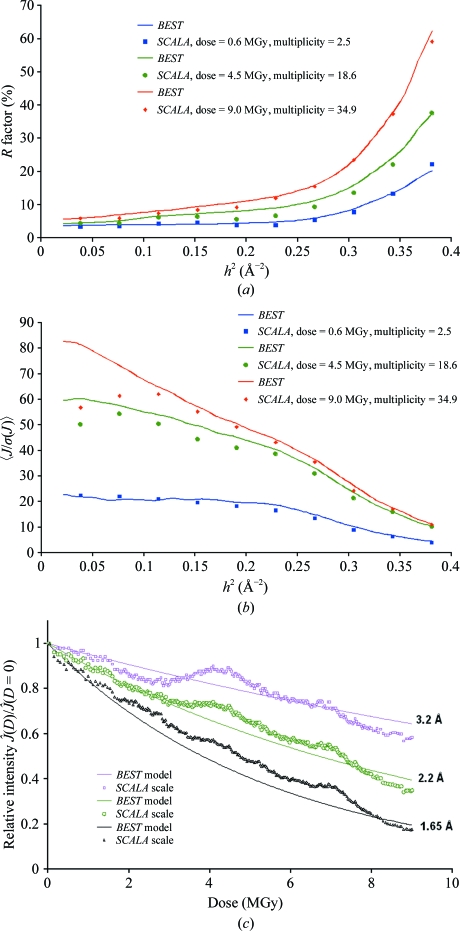
Test-data collection for cubic insulin crystals. (*a*) Predicted and experimental *R*_merge_ *versus* resolution. (*b*) Predicted and experimental 〈*J*/σ(*J*)〉 *versus* resolution. (*c*) Predicted and experimental relative diffraction intensities, 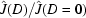.

Small (35 µm) equidimensional bovine insulin crystals (Nanao *et al.*, 2005 ▶) were used for test-data collection. The crystals belonged to space group *I*2_1_3, with unit-cell parameter *a* = 77.9 Å. The incident-beam wavelength was 0.97 Å. The beam was attenuated by a factor of 2. The flux was 1.0 × 10^12^ photons s^−1^ and the estimated dose rate was 0.3 MGy s^−1^. One initial image was measured to 1.5 Å resolution in order to evaluate the crystal quality and to produce the input data for *BEST* modelling, including those presented in Fig. 2 ▶. Subsequently, 300 images were collected with *t*_exp_ = 0.1 s, Δ_ϕ_ = 1° and a resolution of 1.65 Å. Three data sets were obtained after processing and scaling these images. The first data set included the first 20 images and provided a complete (99%) data set with a multiplicity of 2.5 and a low total absorbed dose of 0.6 MGy, the second included 150 images (multiplicity of 18.6 and dose of 4.5 MGy) and the third included all data (multiplicity of 34.9 and dose of 9 MGy). The *R*_merge_ and 〈*J*/σ(*J*)〉 statistics for these data sets are compared with *BEST* predictions in Figs. 3 ▶(*a*) and 3 ▶(*b*), respectively. The example shows that *BEST* can accurately predict the statistical characteristics of data sets over a broad range of absorbed doses. The apparent mismatch of the predicted and observed 〈*J*/σ(*J*)〉 statistics in low-resolution shells arises from unaccounted-for systematic errors that are at the level of <1% of the intensity.

Experimental intensity-decay curves in three resolution shells are compared with the decay model used in *BEST* for statistical predictions in Fig. 3 ▶(*c*). The nonmonotonic character of the experimental curves is clearly a consequence of the combination of a slight mismatch of the crystal size with the vertical beam size and minor miscentring of the sample. Despite a noticeable inconsistency between the model and actual measurement conditions, the statistical predictions are in good agreement with the data.

### 4.2. P19–siRNA

Crystals of viral RNA suppressor P19 in complex with small interfering RNA from tomato bushy stunt virus (P19–siRNA; Ye *et al.*, 2003 ▶) belonged to space group *R*32, with unit-cell parameters *a* = *b* = 90.5, *c* = 148.9 Å. The needle-like shape of the crystals, which were 200–300 µm in length and 25 µm thick, permitted the collection of several data sets from the same crystal by translating an unexposed volume into the beam. The incident-beam wavelength was 0.99 Å.

For the irradiation experiment described in §[Sec sec2.1.1]2.1.1 the flux was 2.75 × 10^12^ photons s^−1^ (dose rate 0.54 MGy s^−1^). A fresh part of the same crystal was used for each data collection (P19–siRNA-1A). During this experiment, the flux was 2.2 × 10^12^ photons s^−1^ (dose rate 0.4 MGy s^−1^). Two initial images were measured with a 1° rotation at 0° and 90° angles, respectively, with an exposure time of 0.1 s and resolution of 2.3 Å. A target value of 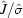 = 2 was set in *BEST*. The strategy calculation showed that a complete data set could be collected to a resolution of 2.45 Å with a total exposure time of 44 s corresponding to a dose of 17.6 MGy. The data-collection strategy is shown in Table 1 ▶; the optimal rotation width was 0.8° for all four subwedges.

**Figure 4:**
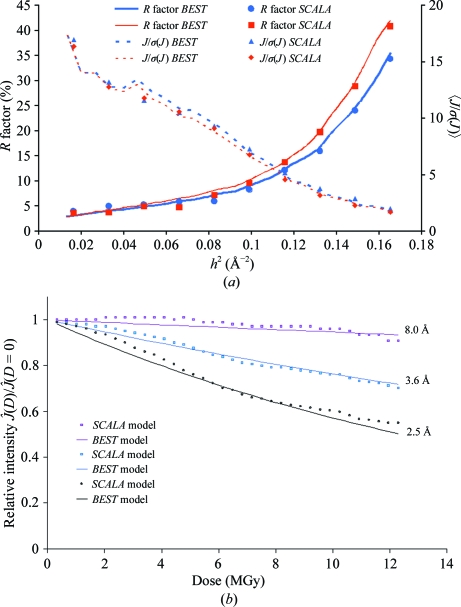
Test-data collection for the P19–siRNA-1 crystal. (*a*) Predicted and experimental *R*_merge_ (solid line) and 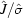 (dashed line) *versus* resolution for P19–siRNA-1A (blue) and P19–siRNA-1B (red). (*b*) Predicted and experimental relative diffraction intensities, 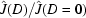, *versus* the dose and resolution for P19–siRNA-1B.

After collecting the P19–siRNA-1 data set, the crystal was recentred on an unexposed part and a second data set, P19–siRNA-1B, was collected using the same starting angle (136°), number of frames (36) and Δ_ϕ_ as for P19–siRNA-1A but with a constant exposure time of 1.22 s, *i.e.* with a total dose equal to that in P19–siRNA-1A. Predicted and calculated data statistics for both data sets are shown by resolution shell in Fig. 4 ▶(*a*); Fig. 4 ▶(*b*) demonstrates how well the *BEST* model describes the diffraction-intensity drop with absorbed dose under close-to-ideal exposure conditions, *i.e.* when the crystal is smaller than the beam in a vertical direction.

Even though the same ‘optimum’ total dose was used for both data sets, the data statistics are noticeably worse for P19–siRNA-1B. The effect of decay compensation by exposure time in P19–siRNA-1A is less pronounced when looking at the spherically averaged 〈*J*/σ(*J*)〉 statistics, which are insensitive with respect to the homogeneity in signal-to-noise distribution within a resolution shell. The significant increase in *R*_merge_ in high-resolution shells is indicative of a severe degradation of the diffracted intensity towards the last frames of P19–siRNA-1B (Fig. 4 ▶ *b*). This was correctly predicted and successfully compensated for by increasing the exposure time of the last frames in P19–siRNA-1A.

In a second experiment, a different more strongly diffracting P19–siRNA crystal was used. The flux was 1.1 × 10^12^ photons s^−1^ and the dose rate was 0.2 MGy s^−1^. An identical initial image-collection procedure (but with the detector distance set to yield a resolution of 2.0 Å) and calculations resulted in a strategy for the P19–siRNA-2A data set (Table 2 ▶) at a resolution of 2.06 Å with a total exposure time of 44 s and a dose of 8.7 MGy.

**Figure 5:**
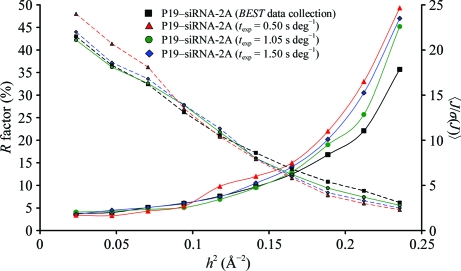
Experimental *R*_merge_ (solid line) and 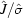 (dashed line) *versus* resolution for test-data sets P19–siRNA-2A (black squares), P19–siRNA-2B (red triangles), P19–siRNA-2C (green circles) and P19–siRNA-2D (blue diamonds).

Next, three further data sets, P19–siRNA-2B, P19–siRNA-2C and P19–siRNA-2D, were collected from the same crystal translated to an unexposed region for each. For these data sets the same rotation range as for P19–siRNA-2A was used (*i.e.* the same starting angle and constant Δ_ϕ_ = 1°; the number of frames was 42). *t*_exp_ was 1.05, 0.5 and 1.5 s for P19–siRNA-2B, P19–siRNA-2C and P19–siRNA-2D, respectively, corresponding to equal total doses for P19–siRNA-2A and P19–siRNA-2B, an approximately 50% lower dose for P19–siRNA-2C and a 50% higher dose for P19–siRNA-2D. The data statistics for all four data sets are compared in Fig. 5 ▶. The statistics of P19–siRNA-2A are clearly better than those of the other data sets in the high-resolution shells.

### 4.3. FAE

Crystals of the feruloyl esterase module of xylanase 10B from *Clostridium thermocellum* (FAE; Prates *et al.*, 2001 ▶) belonged to space group *P*2_1_2_1_2_1_, with unit-cell parameters *a* = 65.4, *b* = 108.8, *c* = 113.9 Å. The ESRF storage ring was operated at only 30 mA current, so the beam flux was only 0.3 × 10^12^ photons s^−1^. The wavelength was 0.99 Å. Two initial images were measured with 1° rotation at 0° and 90° with an exposure time of 0.1 s and a resolution of 1.2 Å at the edge of the detector.

**Figure 6:**
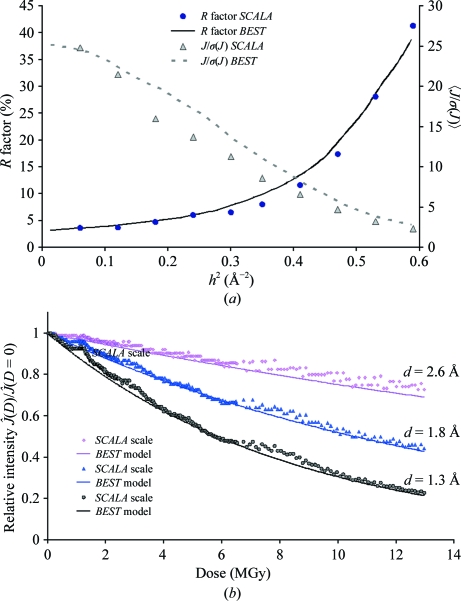
Test-data collection from an FAE crystal. (*a*) Predicted and experimental *R*_merge_ and 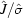 *versus* resolution. (*b*) Predicted and experimental relative diffraction intensity, 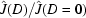, *versus* dose and resolution. The nominal dose rate was 60 kGy s^−1^ (see text for details); this rate multiplied by the cumulative exposure time is used as the dose axis.

In this experiment the crystal size substantially exceeded the beam size. Obviously, under such conditions an essential assumption of the model, namely that at a rotation angle ϕ the diffracting volume receiving the dose *D* = ρ*_D_t*_exp_(ϕ − ϕ_start_)/Δ_ϕ_ (in equation 5) is the same, does not hold as fresh unexposed fractions of the crystal are coming into the beam during rotation. In order to partly compensate for this effect, a dose rate of 24 kGy s^−1^ was used in strategy optimization instead of an estimated nominal (for a static sample) dose rate of 60 kGy s^−1^. This reduces the dose rate by a (fudge) factor of 2.5, which is approximately equal to the ratio of the maximum crystal size in the direction normal to the spindle axis to the vertical FWHM size of the beam. The strategy optimization with a requested 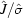 of 2 in the last resolution shell showed that a complete data set could be collected to 1.3 Å with a total exposure time of 217 s (Table 3 ▶). Despite this rather simplistic approach, which may only roughly compensate for the lack of information on the real behaviour of the exposed crystal volume as a function of rotation angle (see §[Sec sec5]5), the predicted and observed data statistics (Fig. 6 ▶ *a*), as well as the predicted and observed intensity-decay curves in resolution shells (Fig. 6 ▶ *b*), agree well.

### 4.4. FtsH

The 70 kDa membrane protein FtsH from *Aquifex aeolicus* crystallizes in space group *I*222, with unit-cell parameters *a* = 137.9, *b* = 162.1, *c* = 170 Å and three FtsH molecules in the asymmetric unit. The crystals grew in 60% Tacsimate pH 7.0 and 10 m*M* AMP-PNP and exhibited moderate diffraction quality. A bipyramidal sample approximately 120 µm in the largest dimension and 50 µm in the smallest dimension was used for data collection at a wavelength of 1.055 Å and a beam flux of 4 × 10^11^ photons s^−1^. The estimated dose rate was nominally 70 kGy s^−1^. In order to exploit nearly the whole crystal volume, the sample position relative to the beam was changed five times during data collection, with a relatively small rotation of 30° used per position.

**Figure 7:**
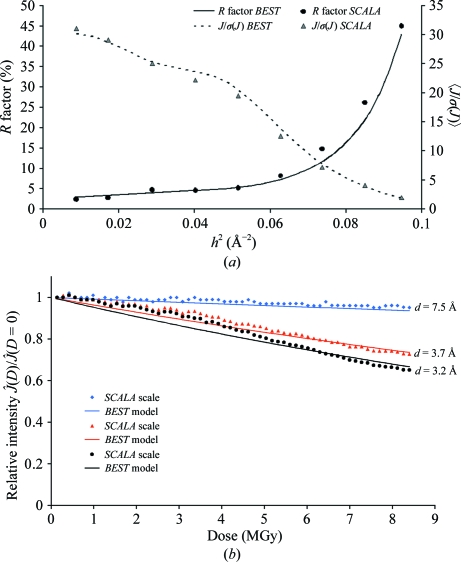
Data collection from an FtsH crystal. (*a*) Predicted and experimental *R*_merge_ and 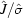 ratio *versus* resolution. (*b*) Predicted and experimental relative diffraction intensity, 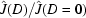, *versus* dose and resolution.

Thus, it appeared possible to collect 150° of data with a multiplicity of about 6. Under these conditions, 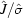  ≃ 3 for the last resolution shell (3.25–3.15 Å) in a complete data set would be reached provided that five 30° data wedges were measured so that 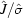  ≃ 1.5 in each of them. The latter was set as a statistical target in the optimization of (constant) exposure time and oscillation width for a 30° wedge starting at 0°. An initial image measured at ϕ = 15° was used in *BEST*. The decay compensation normally achieved by changing the exposure time was disabled, simply because the manual implementation of data collection and processing for a large number of (sub)wedges would have been too tedious to perform and prone to mistakes. Optimization resulted in an achievable resolution limit of 3.15 Å, with *t*_exp_ = 2.0 s and Δ_ϕ_ = 0.50°. For an optimized wedge, the experimental decay curves and the data-processing statistics are in excellent agreement with the data (Figs. 7 ▶ *a* and 7 ▶ *b*). By repeating the same strategy for another four wedges, a complete data set was collected.

Despite the complications, the data set was of good quality (Table 4 ▶) and the data statistics are close to expected values. The structure was solved by molecular replacement a short time after the experiment (Vostrukhina & Baumann, personal communication).

It is worth noting that for this particular example the residual scattering intensity at the end of data collection is ∼65% of the starting value in the last resolution shell (Fig. 7 ▶ *b*), which is a much larger decrease than in all of the other examples (Figs. 3 ▶ *c*, 4 ▶ *b* and 6 ▶ *b*). This is a consequence of the fact that we disabled the facility for changing the exposure time to com­pensate for decay and this example provides a good illustration of the advantages of such compensation. The residual scattering power would still have permitted the collection of more data on the same part of the crystal, suggesting that even longer exposures might have been used to improve the signal-to-noise ratio. As the *BEST* calculations show, this was not the case. For longer exposures the signal to noise would improve only in the first frames of the wedge; it would degrade even more strongly for the last frames and thus degrade overall. The validity of the calculations is in turn directly supported by the experimental data (Fig. 7 ▶ *a*).

## 5. Discussion

Experimenters collecting data on undulator beamlines have been confronted with the dilemma of underexposing *versus* overexposing their samples for a long time. Without a doubt, an educated crystallographer possessing significant experience in data collection on a particular crystal system at a particular instrument would usually find close-to-optimal conditions (*e.g.* similar to those shown in Fig. 5 ▶). Here, we demonstrate that under experimental conditions close to the model assumptions (*i.e.* the instrument is calibrated, the beam size matches the crystal size and the chemical composition of the sample is approximately known) our approach delivers an optimal data-collection strategy in a systematic way. It would be difficult (in our hands, rather impossible) to find notably better strategies.

Furthermore, as the application examples demonstrate, the method is tolerant with respect to the deviations from ideal conditions in real experiments. For instance, in the case of the FAE crystals, which were highly mismatched in size to the beam dimensions, we were able to adapt the model simply by applying a fudge factor to the dose rate. A fudge factor equal to the ratio of the beam size to crystal size is roughly applicable for any space group or redundancy. Such tolerance is directly explained by a very slow variation in signal to noise with the absorbed dose in the vicinity of the maximum (Fig. 2 ▶). This further indicates that the requirements for the accuracy of the flux-density calibration and other parameters involved in the dose calculations are essentially relaxed. As a rule of thumb, ∼20% accurate dose-rate estimates would be sufficient for practical purposes.

Nevertheless, the assumption that the beam size matches the crystal size currently remains a major limitation to the accuracy of the method. In many cases, for example for large plate-like crystals measured in a small beam, the errors in the statistical prediction will be much larger. Here, the data-collection procedures need to employ multiple recentrings or some other manoeuvres similar to those described for the example of FtsH. This application demonstrates that the radiation-damage model-based optimization can be used successfully in more complex scanning diffraction experiments. If a three-dimensional model of the crystal shape and a two-dimensional model of the beam profile were available, further development of the model which could take this information into account appears to be fairly straightforward. For crystal sizes in the range of several tens of micrometres or larger, methods of sample-shape characterizations exist (Leal *et al.*, 2008 ▶; Brockhauser *et al.*, 2008 ▶). Thus, for the range of beam sizes and crystals at a normal macromolecular crystallo­graphy beamline, such as ID23-1 at the ESRF, this development is technically feasible. Extension of the technique to micrometre-sized beam applications (Moukhametzianov *et al.*, 2008 ▶) will be more demanding, but will be justified by the anticipation of a very significant gain in the data quality under the extreme dose rates delivered by the microbeams.

Another limitation to the practical applicability of the method at the beamlines may be related to a certain increase in the complexity of the data-collection procedure. This is largely overcome by software integration, *e.g.* in the *EDNA* on-line data-analysis framework (Incardona *et al.*, 2009 ▶).

The demonstrated tolerance of the method with respect to deviations from ideal model conditions can be extrapolated to the possible variations in radiation-sensitivity between different macromolecular structures. Until now, we have not been confronted with a sample that could confidently be classified as significantly more or significantly less radiation-sensitive compared with the samples described by default model parameters (α and β); in practice, apparent deviations in radiation-sensitivity often do not arise from a specific feature of a crystal structure but rather from a mismatched beam size, mis-calibration or other technical problems. If such an example were to occur, it could be resolved by recalibrating the model in a preliminary experiment involving a sacrificial sample or a part of the sample. The optimization algorithm can easily accommodate a change in the empirical decay constant or, if required, an alternative to the simple exponential model used here.

It is important to note that our radiation-damage model is essentially incomplete and may not be able to exhaustively account for the whole variety of radiation-induced processes occurring in crystals during data collection and their effects on the structure factors. It only accounts for the most pronounced systematic effects, the ‘global’ damage following the terminology of Holton (2008 ▶), and has the sole purpose of optimizing the data collection. ‘Specific’ damage is neglected. The optimization method is geared towards providing data to the highest possible resolution and implies a risk of inducing strong site-specific damage. This may lead in some particular cases to mis-interpretations of the structure. Whenever data on the radiation-sensitivity of a site in question are available, appropriate dose constraints should be used in strategy optimization. Such an option is available in *BEST*. Note that *BEST* optimization will provide the optimum data-collection conditions and also the highest possible resolution in such cases.

A further possible consequence of choosing the last resolution-shell statistics and the resolution limit as optimization targets is that associated low-resolution data may not be collected optimally at the same time. One can see this effect in all the data presented here in Fig. 5 ▶. In this sense, the method described here is only applicable to a range of experiments aiming at data collection to the highest possible resolution but at the limit of statistical significance. Even for such experiments, a separate low-resolution collection run often appears to be useful irrespective of detector overloads. This can easily be planned together with the high-resolution pass and only requires a separate run of *BEST* with an appropriate dose constraint (*e.g.* a small fraction, <10%, of the dose allocated to a high-resolution pass). For experiments aiming at highly accurate data at low to medium resolution, as in an anomalous scattering phasing experiment, the 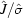 criterion used in this work would not be a suitable optimization target. We have derived a new statistical target specifically for the optimization of SAD data collection that is directly related to the noise in anomalous difference data and have developed methods of optimizing the data collection to this target. A manuscript describing these results is currently in preparation.

The program *BEST* is available for download at http://www.embl-hamburg.de/BEST.

## Figures and Tables

**Table 1:** Data-collection plan for P19–siRNA-1A

ϕ_start_ (°)	No. of images	*t*_exp_ (s)
136.0	19	0.59
151.2	5	1.04
155.2	8	1.75
161.6	4	3.79

**Table 2:** Data-collection strategy for P19–siRNA-2A

ϕ_start_ (°)	No. of images	Δϕ (°)	*t*_exp_ (s)
90.0	18	1.10	0.81
109.8	17	0.85	1.02
124.25	8	0.75	1.47

**Table 3:** Data-collection strategy for FAE

ϕ_start_ (°)	No. of images	Rotation width (°)	Exposure (s)
170.0	120	0.25	0.34
200.0	50	0.50	1.37
225.0	60	0.25	1.79

**Table 4:** Data-processing statistics for FtsH Values in parentheses are for the highest resolution shell

Resolution (Å)	30.00–3.15 (3.25–3.15)
Completeness (%)	99.1 (99.6)
Multiplicity	6.1
〈*J*/σ(*J*)〉	14.7 (2.8)
*R*_merge_ (%)	7.2 (57.3)
